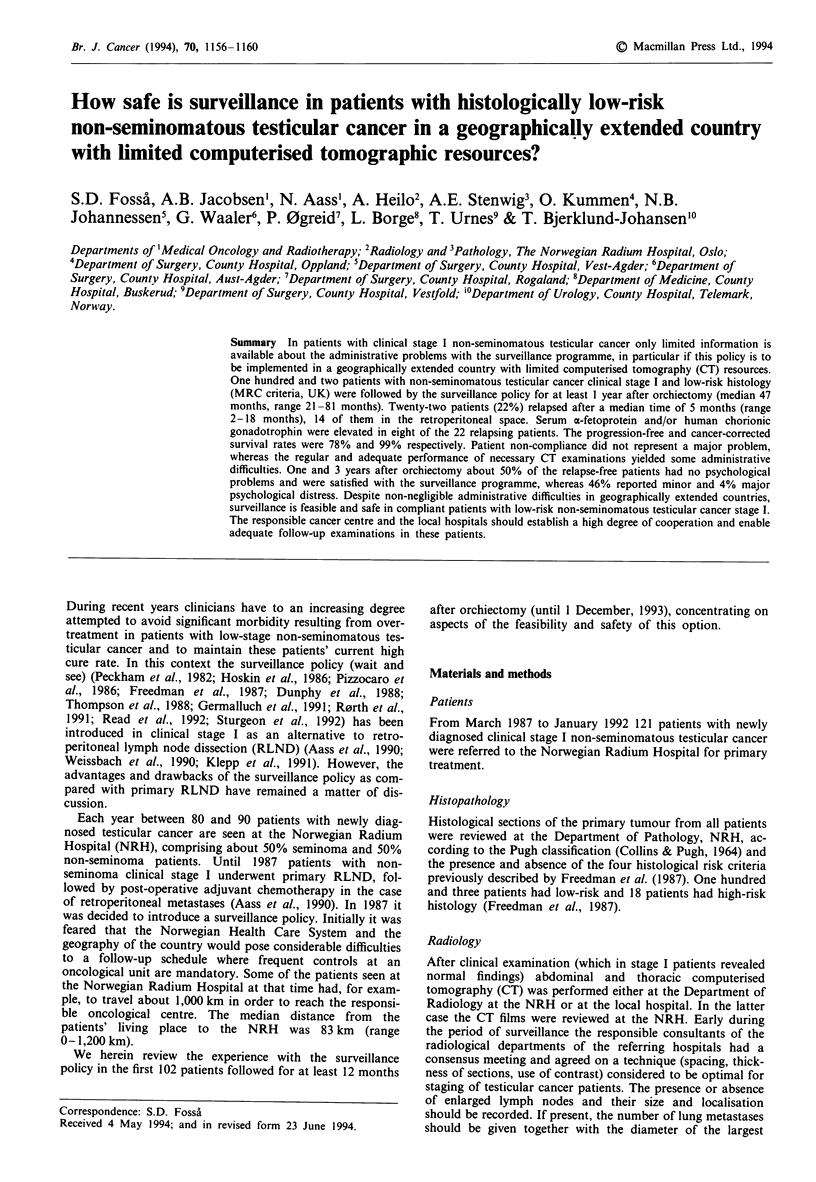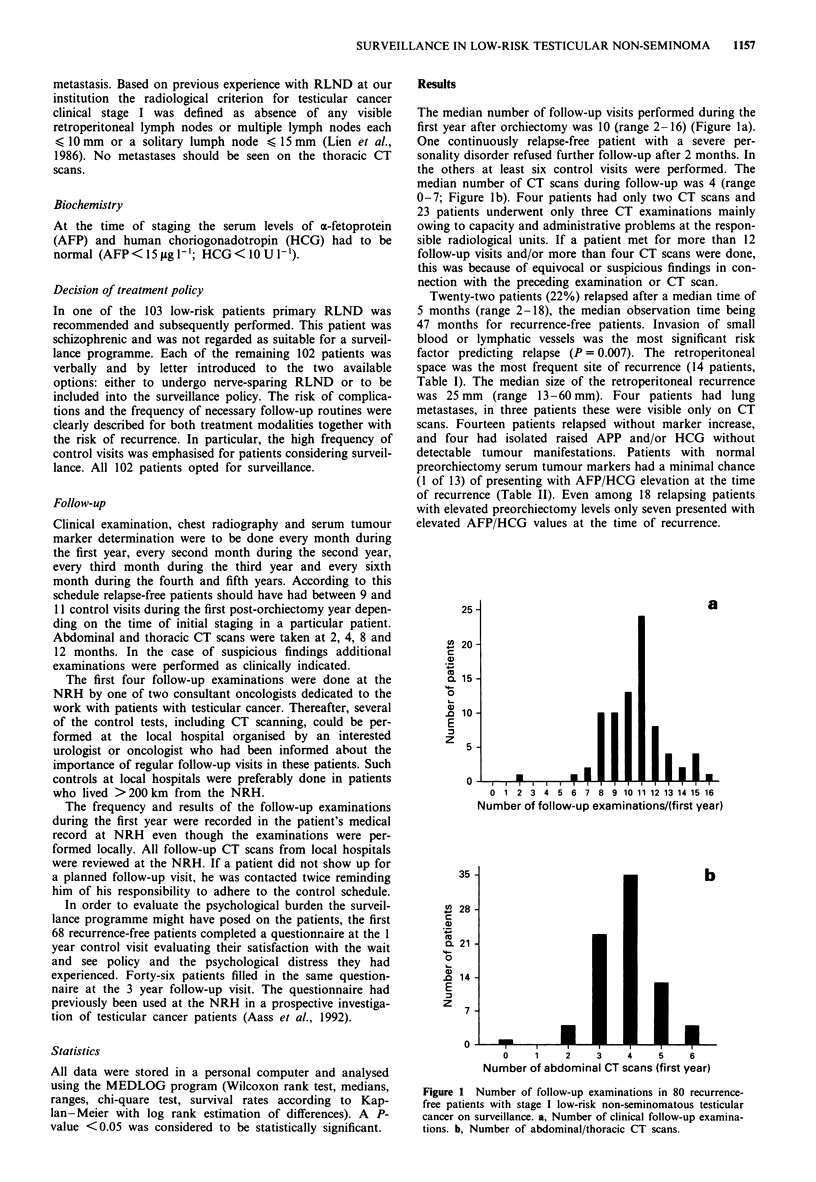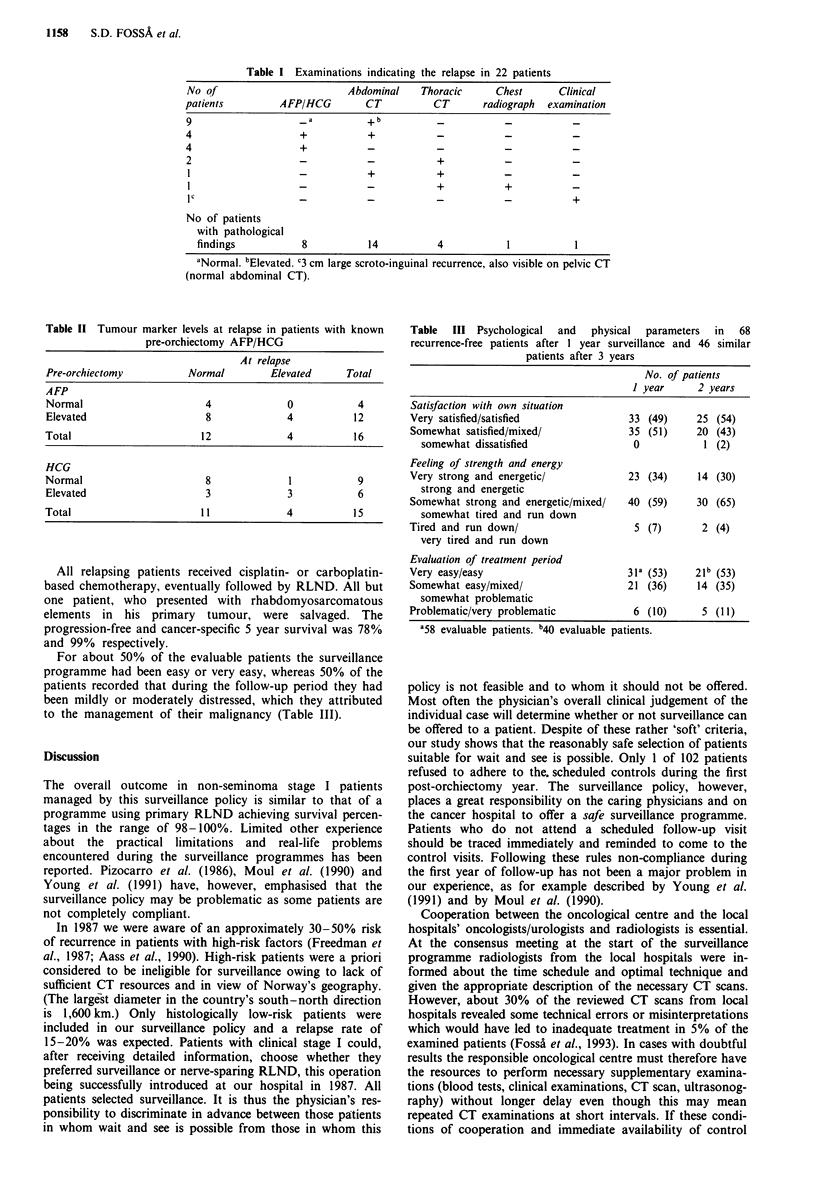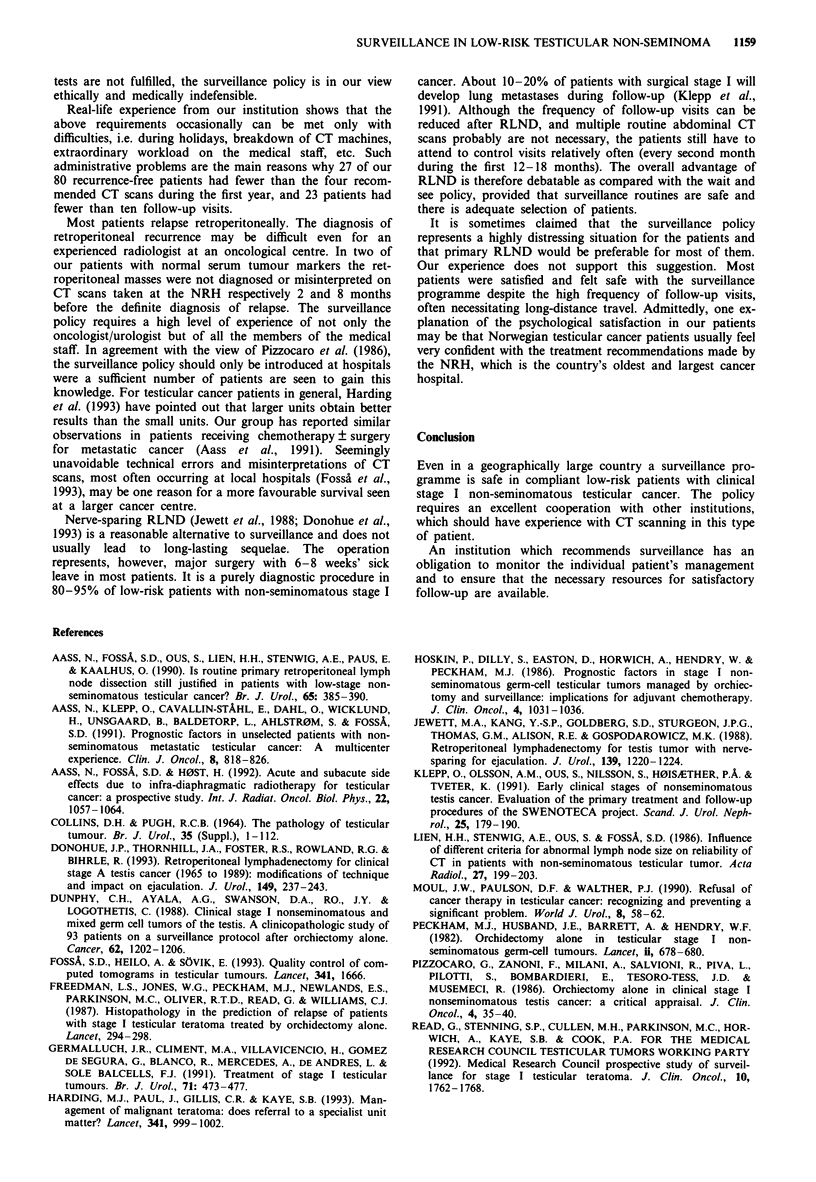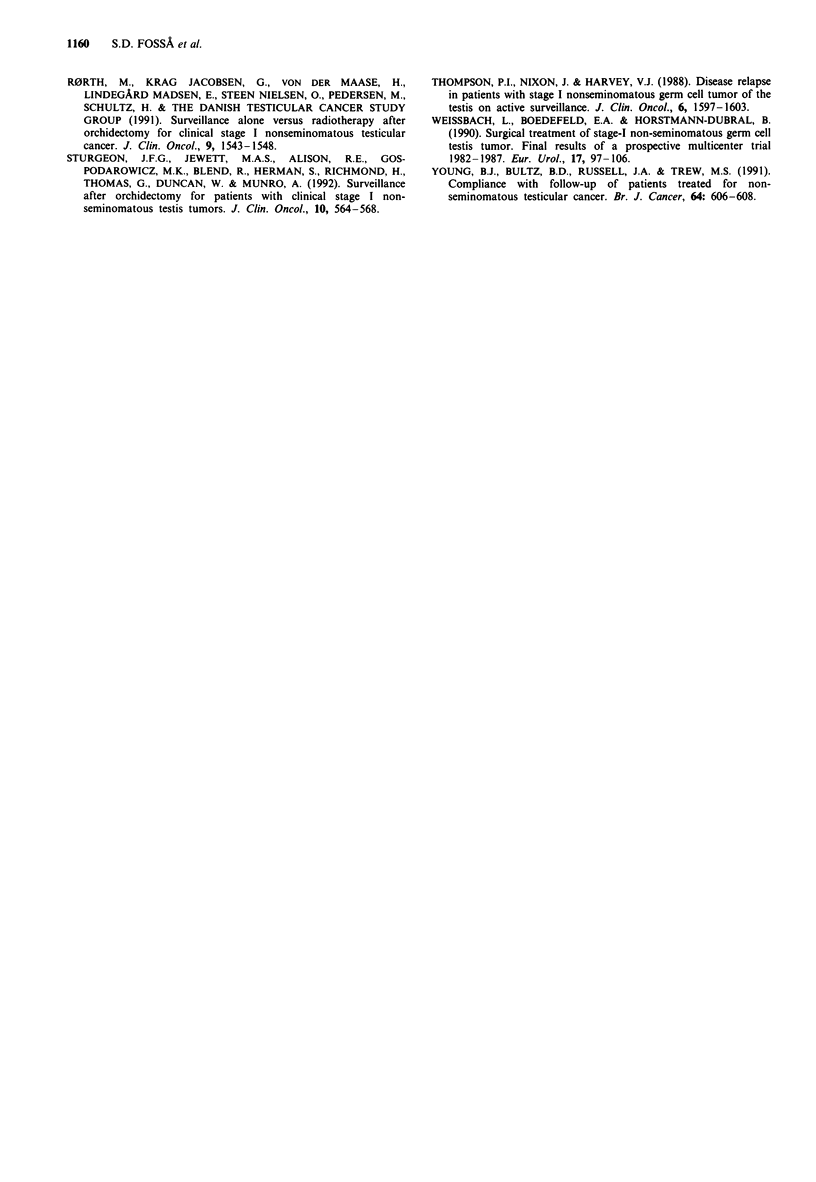# How safe is surveillance in patients with histologically low-risk non-seminomatous testicular cancer in a geographically extended country with limited computerised tomographic resources?

**DOI:** 10.1038/bjc.1994.464

**Published:** 1994-12

**Authors:** S. D. Fosså, A. B. Jacobsen, N. Aass, A. Heilo, A. E. Stenwig, O. Kummen, N. B. Johannessen, G. Waaler, P. Ogreid, L. Borge

**Affiliations:** Department of Medical Oncology, Norwegian Radium Hospital, Oslo.

## Abstract

In patients with clinical stage I non-seminomatous testicular cancer only limited information is available about the administrative problems with the surveillance programme, in particular if this policy is to be implemented in a geographically extended country with limited computerised tomography (CT) resources. One hundred and two patients with non-seminomatous testicular cancer clinical stage I and low-risk histology (MRC criteria, UK) were followed by the surveillance policy for at least 1 year after orchiectomy (median 47 months, range 21-81 months). Twenty-two patients (22%) relapsed after a median time of 5 months (range 2-18 months), 14 of them in the retroperitoneal space. Serum alpha-fetoprotein and/or human chorionic gonadotrophin were elevated in eight of the 22 relapsing patients. The progression-free and cancer-corrected survival rates were 78% and 99% respectively. Patient non-compliance did not represent a major problem, whereas the regular and adequate performance of necessary CT examinations yielded some administrative difficulties. One and 3 years after orchiectomy about 50% of the relapse-free patients had no psychological problems and were satisfied with the surveillance programme, whereas 46% reported minor and 4% major psychological distress. Despite non-negligible administrative difficulties in geographically extended countries, surveillance is feasible and safe in compliant patients with low-risk non-seminomatous testicular cancer stage I. The responsible cancer centre and the local hospitals should establish a high degree of cooperation and enable adequate follow-up examinations in these patients.


Br. J. Cancer (1994), 70, 1156-1160

© Macmillan Press Ltd., 1994

### How safe is surveillance in patients with histologically low-risk non-seminomatous testicular cancer in a geographically extended country with limited computerised tomographic resources?

S.D. Fosså, A.B. Jacobsen[1], N. Aass[1], A. Heilo[2], A.E. Stenwig[3], O. Kummen[4], N.B. Johannessen[5], G. Waaler[6], P. Øgreid[7], L. Borge[8], T. Urnes[9] & T. Bjerklund-Johansen[10]

*Departments of [1]Medical Oncology and Radiotherapy; [2]Radiology and [3]Pathology, The Norwegian Radium Hospital, Oslo; [4]Department of Surgery, County Hospital, Oppland; [5]Department of Surgery, County Hospital, Vest-Agder; [6]Department of Surgery, County Hospital, Aust-Agder; [7]Department of Surgery, County Hospital, Rogaland; [8]Department of Medicine, County Hospital, Buskerud; [9]Department of Surgery, County Hospital, Vestfold; [10]Department of Urology, County Hospital, Telemark, Norway.*

**Summary** In patients with clinical stage I non-seminomatous testicular cancer only limited information is available about the administrative problems with the surveillance programme, in particular if this policy is to be implemented in a geographically extended country with limited computerised tomography (CT) resources. One hundred and two patients with non-seminomatous testicular cancer clinical stage I and low-risk histology (MRC criteria, UK) were followed by the surveillance policy for at least 1 year after orchiectomy (median 47 months, range 21-81 months). Twenty-two patients (22%) relapsed after a median time of 5 months (range 2-18 months), 14 of them in the retroperitoneal space. Serum α-fetoprotein and/or human chorionic gonadotrophin were elevated in eight of the 22 relapsing patients. The progression-free and cancer-corrected survival rates were 78% and 99% respectively. Patient non-compliance did not represent a major problem, whereas the regular and adequate performance of necessary CT examinations yielded some administrative difficulties. One and 3 years after orchiectomy about 50% of the relapse-free patients had no psychological problems and were satisfied with the surveillance programme, whereas 46% reported minor and 4% major psychological distress. Despite non-negligible administrative difficulties in geographically extended countries, surveillance is feasible and safe in compliant patients with low-risk non-seminomatous testicular cancer stage I. The responsible cancer centre and the local hospitals should establish a high degree of cooperation and enable adequate follow-up examinations in these patients.

During recent years clinicians have to an increasing degree attempted to avoid significant morbidity resulting from overtreatment in patients with low-stage non-seminomatous testicular cancer and to maintain these patients' current high cure rate. In this context the surveillance policy (wait and see) (Peckham *et al.*, 1982; Hoskin *et al.*, 1986; Pizzocaro *et al.*, 1986; Freedman *et al.*, 1987; Dunphy *et al.*, 1988; Thompson *et al.*, 1988; Germalluch *et al.*, 1991; Rørth *et al.*, 1991; Read *et al.*, 1992; Sturgeon *et al.*, 1992) has been introduced in clinical stage I as an alternative to retroperitoneal lymph node dissection (RLND) (Aass *et al.*, 1990; Weissbach *et al.*, 1990; Klepp *et al.*, 1991). However, the advantages and drawbacks of the surveillance policy as compared with primary RLND have remained a matter of discussion.

Each year between 80 and 90 patients with newly diagnosed testicular cancer are seen at the Norwegian Radium Hospital (NRH), comprising about 50% seminoma and 50% non-seminoma patients. Until 1987 patients with non-seminoma clinical stage I underwent primary RLND, followed by post-operative adjuvant chemotherapy in the case of retroperitoneal metastases (Aass *et al.*, 1990). In 1987 it was decided to introduce a surveillance policy. Initially it was feared that the Norwegian Health Care System and the geography of the country would pose considerable difficulties to a follow-up schedule where frequent controls at an oncological unit are mandatory. Some of the patients seen at the Norwegian Radium Hospital at that time had, for example, to travel about 1,000 km in order to reach the responsible oncological centre. The median distance from the patients' living place to the NRH was 83 km (range 0-1,200 km).

We herein review the experience with the surveillance policy in the first 102 patients followed for at least 12 months after orchiectomy (until 1 December, 1993), concentrating on aspects of the feasibility and safety of this option.

Correspondence: S.D. Fosså

Received 4 May 1994; and in revised form 23 June 1994.

### Materials and methods

### *Patients*

From March 1987 to January 1992 121 patients with newly diagnosed clinical stage I non-seminomatous testicular cancer were referred to the Norwegian Radium Hospital for primary treatment.

### *Histopathology*

Histological sections of the primary tumour from all patients were reviewed at the Department of Pathology, NRH, according to the Pugh classification (Collins & Pugh, 1964) and the presence and absence of the four histological risk criteria previously described by Freedman *et al.* (1987). One hundred and three patients had low-risk and 18 patients had high-risk histology (Freedman *et al.*, 1987).

### *Radiology*

After clinical examination (which in stage I patients revealed normal findings) abdominal and thoracic computerised tomography (CT) was performed either at the Department of Radiology at the NRH or at the local hospital. In the latter case the CT films were reviewed at the NRH. Early during the period of surveillance the responsible consultants of the radiological departments of the referring hospitals had a consensus meeting and agreed on a technique (spacing, thickness of sections, use of contrast) considered to be optimal for staging of testicular cancer patients. The presence or absence of enlarged lymph nodes and their size and localisation should be recorded. If present, the number of lung metastases should be given together with the diameter of the largest metastasis. Based on previous experience with RLND at our institution the radiological criterion for testicular cancer clinical stage I was defined as absence of any visible retroperitoneal lymph nodes or multiple lymph nodes each $\leqslant 10$ mm or a solitary lumph node $\leqslant 15$ mm (Lien *et al.*, 1986). No metastases should be seen on the thoracic CT scans.

*Biochemistry*

At the time of staging the serum levels of α-fetoprotein (AFP) and human choriogonadotropin (HCG) had to be normal (AFP $< 15\ \mu g\ l^{-1}$; HCG $< 10\ U\ l^{-1}$).

*Decision of treatment policy*

In one of the 103 low-risk patients primary RLND was recommended and subsequently performed. This patient was schizophrenic and was not regarded as suitable for a surveillance programme. Each of the remaining 102 patients was verbally and by letter introduced to the two available options: either to undergo nerve-sparing RLND or to be included into the surveillance policy. The risk of complications and the frequency of necessary follow-up routines were clearly described for both treatment modalities together with the risk of recurrence. In particular, the high frequency of control visits was emphasised for patients considering surveillance. All 102 patients opted for surveillance.

*Follow-up*

Clinical examination, chest radiography and serum tumour marker determination were to be done every month during the first year, every second month during the second year, every third month during the third year and every sixth month during the fourth and fifth years. According to this schedule relapse-free patients should have had between 9 and 11 control visits during the first post-orchiectomy year depending on the time of initial staging in a particular patient. Abdominal and thoracic CT scans were taken at 2, 4, 8 and 12 months. In the case of suspicious findings additional examinations were performed as clinically indicated.

The first four follow-up examinations were done at the NRH by one of two consultant oncologists dedicated to the work with patients with testicular cancer. Thereafter, several of the control tests, including CT scanning, could be performed at the local hospital organised by an interested urologist or oncologist who had been informed about the importance of regular follow-up visits in these patients. Such controls at local hospitals were preferably done in patients who lived $>200$ km from the NRH.

The frequency and results of the follow-up examinations during the first year were recorded in the patient's medical record at NRH even though the examinations were performed locally. All follow-up CT scans from local hospitals were reviewed at the NRH. If a patient did not show up for a planned follow-up visit, he was contacted twice reminding him of his responsibility to adhere to the control schedule.

In order to evaluate the psychological burden the surveillance programme might have posed on the patients, the first 68 recurrence-free patients completed a questionnaire at the 1 year control visit evaluating their satisfaction with the wait and see policy and the psychological distress they had experienced. Forty-six patients filled in the same questionnaire at the 3 year follow-up visit. The questionnaire had previously been used at the NRH in a prospective investigation of testicular cancer patients (Aass *et al.*, 1992).

*Statistics*

All data were stored in a personal computer and analysed using the MEDLOG program (Wilcoxon rank test, medians, ranges, chi-quare test, survival rates according to Kaplan–Meier with log rank estimation of differences). A $P$-value $<0.05$ was considered to be statistically significant.

### Results

The median number of follow-up visits performed during the first year after orchiectomy was 10 (range 2–16) (Figure 1a). One continuously relapse-free patient with a severe personality disorder refused further follow-up after 2 months. In the others at least six control visits were performed. The median number of CT scans during follow-up was 4 (range 0–7; Figure 1b). Four patients had only two CT scans and 23 patients underwent only three CT examinations mainly owing to capacity and administrative problems at the responsible radiological units. If a patient met for more than 12 follow-up visits and/or more than four CT scans were done, this was because of equivocal or suspicious findings in connection with the preceding examination or CT scan.

Twenty-two patients (22%) relapsed after a median time of 5 months (range 2–18), the median observation time being 47 months for recurrence-free patients. Invasion of small blood or lymphatic vessels was the most significant risk factor predicting relapse ($P = 0.007$). The retroperitoneal space was the most frequent site of recurrence (14 patients, Table I). The median size of the retroperitoneal recurrence was 25 mm (range 13–60 mm). Four patients had lung metastases, in three patients these were visible only on CT scans. Fourteen patients relapsed without marker increase, and four had isolated raised APP and/or HCG without detectable tumour manifestations. Patients with normal preorchiectomy serum tumour markers had a minimal chance (1 of 13) of presenting with AFP/HCG elevation at the time of recurrence (Table II). Even among 18 relapsing patients with elevated preorchiectomy levels only seven presented with elevated AFP/HCG values at the time of recurrence.

**Figure 1** Number of follow-up examinations in 80 recurrence-free patients with stage I low-risk non-seminomatous testicular cancer on surveillance. **a**, Number of clinical follow-up examinations. **b**, Number of abdominal/thoracic CT scans.

**Table I** Examinations indicating the relapse in 22 patients

| *No of patients* | *AFP/HCG* | *Abdominal CT* | *Thoracic CT* | *Chest radiograph* | *Clinical examination* |
|---|---|---|---|---|---|
| 9 | –[a] | +[b] | – | – | – |
| 4 | + | + | – | – | – |
| 4 | + | – | – | – | – |
| 2 | – | – | + | – | – |
| 1 | – | + | + | – | – |
| 1 | – | – | + | + | – |
| 1[c] | – | – | – | – | + |
| No of patients with pathological findings | 8 | 14 | 4 | 1 | 1 |

[a]Normal. [b]Elevated. [c]3 cm large scroto-inguinal recurrence, also visible on pelvic CT (normal abdominal CT).

**Table II** Tumour marker levels at relapse in patients with known pre-orchiectomy AFP/HCG

| *Pre-orchiectomy* | *At relapse* Normal | Elevated | *Total* |
|---|---|---|---|
| *AFP* | | | |
| Normal | 4 | 0 | 4 |
| Elevated | 8 | 4 | 12 |
| Total | 12 | 4 | 16 |
| *HCG* | | | |
| Normal | 8 | 1 | 9 |
| Elevated | 3 | 3 | 6 |
| Total | 11 | 4 | 15 |

**Table III** Psychological and physical parameters in 68 recurrence-free patients after 1 year surveillance and 46 similar patients after 3 years

| | *No. of patients* 1 year | 2 years |
|---|---|---|
| *Satisfaction with own situation* | | |
| Very satisfied/satisfied | 33 (49) | 25 (54) |
| Somewhat satisfied/mixed/ somewhat dissatisfied | 35 (51) | 20 (43) |
| | 0 | 1 (2) |
| *Feeling of strength and energy* | | |
| Very strong and energetic/ strong and energetic | 23 (34) | 14 (30) |
| Somewhat strong and energetic/mixed/ somewhat tired and run down | 40 (59) | 30 (65) |
| Tired and run down/ very tired and run down | 5 (7) | 2 (4) |
| *Evaluation of treatment period* | | |
| Very easy/easy | 31[a] (53) | 21[b] (53) |
| Somewhat easy/mixed/ somewhat problematic | 21 (36) | 14 (35) |
| Problematic/very problematic | 6 (10) | 5 (11) |

[a]58 evaluable patients. [b]40 evaluable patients.

All relapsing patients received cisplatin- or carboplatin-based chemotherapy, eventually followed by RLND. All but one patient, who presented with rhabdomyosarcomatous elements in his primary tumour, were salvaged. The progression-free and cancer-specific 5 year survival was 78% and 99% respectively.

For about 50% of the evaluable patients the surveillance programme had been easy or very easy, whereas 50% of the patients recorded that during the follow-up period they had been mildly or moderately distressed, which they attributed to the management of their malignancy (Table III).

### Discussion

The overall outcome in non-seminoma stage I patients managed by this surveillance policy is similar to that of a programme using primary RLND achieving survival percentages in the range of 98–100%. Limited other experience about the practical limitations and real-life problems encountered during the surveillance programmes has been reported. Pizocarro *et al.* (1986), Moul *et al.* (1990) and Young *et al.* (1991) have, however, emphasised that the surveillance policy may be problematic as some patients are not completely compliant.

In 1987 we were aware of an approximately 30–50% risk of recurrence in patients with high-risk factors (Freedman *et al.*, 1987; Aass *et al.*, 1990). High-risk patients were a priori considered to be ineligible for surveillance owing to lack of sufficient CT resources and in view of Norway's geography. (The largest diameter in the country's south–north direction is 1,600 km.) Only histologically low-risk patients were included in our surveillance policy and a relapse rate of 15–20% was expected. Patients with clinical stage I could, after receiving detailed information, choose whether they preferred surveillance or nerve-sparing RLND, this operation being successfully introduced at our hospital in 1987. All patients selected surveillance. It is thus the physician's responsibility to discriminate in advance between those patients in whom wait and see is possible from those in whom this policy is not feasible and to whom it should not be offered. Most often the physician's overall clinical judgement of the individual case will determine whether or not surveillance can be offered to a patient. Despite of these rather 'soft' criteria, our study shows that the reasonably safe selection of patients suitable for wait and see is possible. Only 1 of 102 patients refused to adhere to the scheduled controls during the first post-orchiectomy year. The surveillance policy, however, places a great responsibility on the caring physicians and on the cancer hospital to offer a *safe* surveillance programme. Patients who do not attend a scheduled follow-up visit should be traced immediately and reminded to come to the control visits. Following these rules non-compliance during the first year of follow-up has not been a major problem in our experience, as for example described by Young *et al.* (1991) and by Moul *et al.* (1990).

Cooperation between the oncological centre and the local hospitals' oncologists/urologists and radiologists is essential. At the consensus meeting at the start of the surveillance programme radiologists from the local hospitals were informed about the time schedule and optimal technique and given the appropriate description of the necessary CT scans. However, about 30% of the reviewed CT scans from local hospitals revealed some technical errors or misinterpretations which would have led to inadequate treatment in 5% of the examined patients (Fosså *et al.*, 1993). In cases with doubtful results the responsible oncological centre must therefore have the resources to perform necessary supplementary examinations (blood tests, clinical examinations, CT scan, ultrasonography) without longer delay even though this may mean repeated CT examinations at short intervals. If these conditions of cooperation and immediate availability of control tests are not fulfilled, the surveillance policy is in our view ethically and medically indefensible.

Real-life experience from our institution shows that the above requirements occasionally can be met only with difficulties, i.e. during holidays, breakdown of CT machines, extraordinary workload on the medical staff, etc. Such administrative problems are the main reasons why 27 of our 80 recurrence-free patients had fewer than the four recommended CT scans during the first year, and 23 patients had fewer than ten follow-up visits.

Most patients relapse retroperitoneally. The diagnosis of retroperitoneal recurrence may be difficult even for an experienced radiologist at an oncological centre. In two of our patients with normal serum tumour markers the retroperitoneal masses were not diagnosed or misinterpreted on CT scans taken at the NRH respectively 2 and 8 months before the definite diagnosis of relapse. The surveillance policy requires a high level of experience of not only the oncologist/urologist but of all the members of the medical staff. In agreement with the view of Pizzocaro *et al.* (1986), the surveillance policy should only be introduced at hospitals were a sufficient number of patients are seen to gain this knowledge. For testicular cancer patients in general, Harding *et al.* (1993) have pointed out that larger units obtain better results than the small units. Our group has reported similar observations in patients receiving chemotherapy ± surgery for metastatic cancer (Aass *et al.*, 1991). Seemingly unavoidable technical errors and misinterpretations of CT scans, most often occurring at local hospitals (Fosså *et al.*, 1993), may be one reason for a more favourable survival seen at a larger cancer centre.

Nerve-sparing RLND (Jewett *et al.*, 1988; Donohue *et al.*, 1993) is a reasonable alternative to surveillance and does not usually lead to long-lasting sequelae. The operation represents, however, major surgery with 6–8 weeks' sick leave in most patients. It is a purely diagnostic procedure in 80–95% of low-risk patients with non-seminomatous stage I cancer. About 10–20% of patients with surgical stage I will develop lung metastases during follow-up (Klepp *et al.*, 1991). Although the frequency of follow-up visits can be reduced after RLND, and multiple routine abdominal CT scans probably are not necessary, the patients still have to attend to control visits relatively often (every second month during the first 12–18 months). The overall advantage of RLND is therefore debatable as compared with the wait and see policy, provided that surveillance routines are safe and there is adequate selection of patients.

It is sometimes claimed that the surveillance policy represents a highly distressing situation for the patients and that primary RLND would be preferable for most of them. Our experience does not support this suggestion. Most patients were satisfied and felt safe with the surveillance programme despite the high frequency of follow-up visits, often necessitating long-distance travel. Admittedly, one explanation of the psychological satisfaction in our patients may be that Norwegian testicular cancer patients usually feel very confident with the treatment recommendations made by the NRH, which is the country's oldest and largest cancer hospital.

### Conclusion

Even in a geographically large country a surveillance programme is safe in compliant low-risk patients with clinical stage I non-seminomatous testicular cancer. The policy requires an excellent cooperation with other institutions, which should have experience with CT scanning in this type of patient.

An institution which recommends surveillance has an obligation to monitor the individual patient's management and to ensure that the necessary resources for satisfactory follow-up are available.

### References

AASS, N., FOSSÅ, S.D., OUS, S., LIEN, H.H., STENWIG, A.E., PAUS, E. & KAALHUS, O. (1990). Is routine primary retroperitoneal lymph node dissection still justified in patients with low-stage non-seminomatous testicular cancer? *Br. J. Urol.*, **65**: 385–390.

AASS, N., KLEPP, O., CAVALLIN-STÅHL, E., DAHL, O., WICKLUND, H., UNSGAARD, B., BALDETORP, L., AHLSTRØM, S. & FOSSÅ, S.D. (1991). Prognostic factors in unselected patients with non-seminomatous metastatic testicular cancer: A multicenter experience. *Clin. J. Oncol.*, **8**, 818–826.

AASS, N., FOSSÅ, S.D. & HØST, H. (1992). Acute and subacute side effects due to infra-diaphragmatic radiotherapy for testicular cancer: a prospective study. *Int. J. Radiat. Oncol. Biol. Phys.*, **22**, 1057–1064.

COLLINS, D.H. & PUGH, R.C.B. (1964). The pathology of testicular tumour. *Br. J. Urol.*, **35** (Suppl.), 1–112.

DONOHUE, J.P., THORNHILL, J.A., FOSTER, R.S., ROWLAND, R.G. & BIHRLE, R. (1993). Retroperitoneal lymphadenectomy for clinical stage A testis cancer (1965 to 1989): modifications of technique and impact on ejaculation. *J. Urol.*, **149**, 237–243.

DUNPHY, C.H., AYALA, A.G., SWANSON, D.A., RO., J.Y. & LOGOTHETIS, C. (1988). Clinical stage I nonseminomatous and mixed germ cell tumors of the testis. A clinicopathologic study of 93 patients on a surveillance protocol after orchiectomy alone. *Cancer*, **62**, 1202–1206.

FOSSÅ, S.D., HEILO, A. & SÖVIK, E. (1993). Quality control of computed tomograms in testicular tumours. *Lancet*, **341**, 1666.

FREEDMAN, L.S., JONES, W.G., PECKHAM, M.J., NEWLANDS, E.S., PARKINSON, M.C., OLIVER, R.T.D., READ, G. & WILLIAMS, C.J. (1987). Histopathology in the prediction of relapse of patients with stage I testicular teratoma treated by orchidectomy alone. *Lancet*, 294–298.

GERMALLUCH, J.R., CLIMENT, M.A., VILLAVICENCIO, H., GOMEZ DE SEGURA, G., BLANCO, R., MERCEDES, A., DE ANDRES, L. & SOLE BALCELLS, F.J. (1991). Treatment of stage I testicular tumours. *Br. J. Urol.*, **71**: 473–477.

HARDING, M.J., PAUL, J., GILLIS, C.R. & KAYE, S.B. (1993). Management of malignant teratoma: does referral to a specialist unit matter? *Lancet*, **341**, 999–1002.

HOSKIN, P., DILLY, S., EASTON, D., HORWICH, A., HENDRY, W. & PECKHAM, M.J. (1986). Prognostic factors in stage I non-seminomatous germ-cell testicular tumors managed by orchiectomy and surveillance: implications for adjuvant chemotherapy. *J. Clin. Oncol.*, **4**, 1031–1036.

JEWETT, M.A., KANG, Y.-S.P., GOLDBERG, S.D., STURGEON, J.P.G., THOMAS, G.M., ALISON, R.E. & GOSPODAROWICZ, M.K. (1988). Retroperitoneal lymphadenectomy for testis tumor with nerve-sparing for ejaculation. *J. Urol.*, **139**, 1220–1224.

KLEPP, O., OLSSON, A.M., OUS, S., NILSSON, S., HØISÆTHER, P.Å. & TVETER, K. (1991). Early clinical stages of nonseminomatous testis cancer. Evaluation of the primary treatment and follow-up procedures of the SWENOTECA project. *Scand. J. Urol. Nephrol.*, **25**, 179–190.

LIEN, H.H., STENWIG, A.E., OUS, S. & FOSSÅ, S.D. (1986). Influence of different criteria for abnormal lymph node size on reliability of CT in patients with non-seminomatous testicular tumor. *Acta Radiol.*, **27**, 199–203.

MOUL, J.W., PAULSON, D.F. & WALTHER, P.J. (1990). Refusal of cancer therapy in testicular cancer: recognizing and preventing a significant problem. *World J. Urol.*, **8**, 58–62.

PECKHAM, M.J., HUSBAND, J.E., BARRETT, A. & HENDRY, W.F. (1982). Orchidectomy alone in testicular stage I non-seminomatous germ-cell tumours. *Lancet*, **ii**, 678–680.

PIZZOCARO, G., ZANONI, F., MILANI, A., SALVIONI, R., PIVA, L., PILOTTI, S., BOMBARDIERI, E., TESORO-TESS, J.D. & MUSEMECI, R. (1986). Orchiectomy alone in clinical stage I nonseminomatous testis cancer: a critical appraisal. *J. Clin. Oncol.*, **4**, 35–40.

READ, G., STENNING, S.P., CULLEN, M.H., PARKINSON, M.C., HORWICH, A., KAYE, S.B. & COOK, P.A. FOR THE MEDICAL RESEARCH COUNCIL TESTICULAR TUMORS WORKING PARTY (1992). Medical Research Council prospective study of surveillance for stage I testicular teratoma. *J. Clin. Oncol.*, **10**, 1762–1768.

RØRTH, M., KRAG JACOBSEN, G., VON DER MAASE, H., LINDEGÅRD MADSEN, E., STEEN NIELSEN, O., PEDERSEN, M., SCHULTZ, H. & THE DANISH TESTICULAR CANCER STUDY GROUP (1991). Surveillance alone versus radiotherapy after orchidectomy for clinical stage I nonseminomatous testicular cancer. *J. Clin. Oncol.*, **9**, 1543-1548.

STURGEON, J.F.G., JEWETT, M.A.S., ALISON, R.E., GOSPODAROWICZ, M.K., BLEND, R., HERMAN, S., RICHMOND, H., THOMAS, G., DUNCAN, W. & MUNRO, A. (1992). Surveillance after orchidectomy for patients with clinical stage I nonseminomatous testis tumors. *J. Clin. Oncol.*, **10**, 564-568.

THOMPSON, P.I., NIXON, J. & HARVEY, V.J. (1988). Disease relapse in patients with stage I nonseminomatous germ cell tumor of the testis on active surveillance. *J. Clin. Oncol.*, **6**, 1597-1603.

WEISSBACH, L., BOEDEFELD, E.A. & HORSTMANN-DUBRAL, B. (1990). Surgical treatment of stage-I non-seminomatous germ cell testis tumor. Final results of a prospective multicenter trial 1982-1987. *Eur. Urol.*, **17**, 97-106.

YOUNG, B.J., BULTZ, B.D., RUSSELL, J.A. & TREW, M.S. (1991). Compliance with follow-up of patients treated for non-seminomatous testicular cancer. *Br. J. Cancer*, **64**: 606-608.